# Operationalizing a Rideshare Intervention for Colonoscopy Completion: Barriers, Facilitators, and Process Recommendations

**DOI:** 10.3389/frhs.2021.799816

**Published:** 2022-01-17

**Authors:** Ari Bell-Brown, Lisa Chew, Bryan J. Weiner, Lisa Strate, Bryan Balmadrid, Cara C. Lewis, Peggy Hannon, John M. Inadomi, Scott D. Ramsey, Rachel B. Issaka

**Affiliations:** ^1^Fred Hutchinson Cancer Research Center, Hutchinson Institute for Cancer Outcomes Research, Seattle, WA, United States; ^2^Department of Internal Medicine, University of Washington School of Medicine, Seattle, WA, United States; ^3^Department of Health Systems and Population Health, University of Washington School of Public Health, Seattle, WA, United States; ^4^Division of Gastroenterology, University of Washington School of Medicine, Seattle, WA, United States; ^5^Kaiser Permanente Washington Health Research Institute, Seattle, WA, United States; ^6^Department of Medicine, University of Utah School of Medicine, Salt Lake City, UT, United States; ^7^Clinical Research Division, Fred Hutchinson Cancer Research Center, Seattle, WA, United States

**Keywords:** colorectal cancer, colonoscopy, non-emergency medical transportation, screening, rideshare

## Abstract

**Introduction:**

Transportation is a common barrier to colonoscopy completion for colorectal cancer (CRC) screening. The study aims to identify the barriers, facilitators, and process recommendations to implement a rideshare non-emergency medical transportation (NEMT) intervention following colonoscopy completion within a safety-net healthcare setting.

**Methods:**

We used informal stakeholder engagement, story boards—a novel user-centered design technique, listening sessions and the nominal group technique to identify the barriers, facilitators, and process to implementing a rideshare NEMT program following colonoscopy completion in a large safety-net healthcare system.

**Results:**

Barriers to implementing a rideshare NEMT intervention for colonoscopy completion included: inability to expand an existing NEMT program beyond Medicaid patients and lack of patient chaperones with rideshare NEMT programs. Facilitators included: commercially available rideshare NEMT platforms that were lower cost and had shorter wait times than the alternative of taxis. Operationalizing and implementing a rideshare NEMT intervention in our healthcare system required the following steps: 1) identifying key stakeholders, 2) engaging stakeholder groups in discussion to identify barriers and solutions, 3) obtaining institutional sign-off, 4) developing a process for reviewing and selecting a rideshare NEMT program, 5) executing contracts, 6) developing a standard operating procedure and 7) training clinic staff to use the rideshare platform.

**Discussion:**

Rideshare NEMT after procedural sedation is administered may improve colonoscopy completion rates and provide one solution to inadequate CRC screening. If successful, our rideshare model could be broadly applicable to other safety-net health systems, populations with high social needs, and settings where procedural sedation is administered.

## Introduction

Colorectal Cancer (CRC) is the second leading cause of cancer-deaths in the U.S. (1). There is clear evidence that screening by stool-based tests is cost-effective (2) and saves lives (3); however, screening is underutilized, especially among racial/ethnic minorities and low-income populations (4). In safety-net healthcare settings and federally qualified health centers (FQHC's), where many medically underserved populations receive care, CRC screening improves when fecal immunochemical test (FIT) is offered alongside colonoscopy (5). Due to patient preference and limited resources (6), FIT is a cornerstone for CRC screening in these settings. Among patients with an abnormal FIT result, a missed or delayed diagnostic colonoscopy increases CRC incidence and mortality (7–10). Despite these concerns, the proportion of patients with an abnormal FIT result who complete a diagnostic colonoscopy rarely exceeds 50% in most safety-net systems and FQHCs (11–13).

Lack of patient transportation is a frequently reported logistical barrier to initial colonoscopy completion (14, 15), affecting up to 41% of individuals referred for CRC screening (16), and also contributes to inadequate colonoscopy completion after an abnormal FIT result (17). In the U.S., most endoscopy units require patients have a chaperone who can drive them home after any procedure that uses sedation, including colonoscopy. This poses a significant barrier to CRC screening for patients who lack relatives or friends who can take substantial time off from work. While other barriers to CRC screening exist (e.g., access to care, bowel preparation challenges, etc.), lack of transportation is a priority area to address given its pervasiveness, ubiquity, and chronicity (18).

Major rideshare companies have developed non-emergency medical transportation (NEMT) services that can be scheduled by the healthcare team through a Health Insurance Portability and Accountability Act (HIPAA) compliant platform, costs are billed directly to the organization, and use does not require patients' own a smartphone. Therefore, rideshare NEMT offers a potentially scalable and cost-effective intervention to improve CRC screening by increasing initial and follow-up colonoscopy completion. Evaluations of rideshare NEMT programs have shown initial success in primary care (19) and radiology settings (20), but rideshare NEMT has not been adopted by our healthcare system and has not been evaluated as a post-procedure transportation option in settings where sedation is administered (e.g., endoscopy). Therefore, the aims of this study were to: 1) identify the barriers and facilitators to implementing a rideshare NEMT intervention after colonoscopy completion and 2) outline process recommendations for operationalizing and implementing a rideshare intervention for settings where procedural sedation is administered.

## Methods

Our research took place at Harborview Medical Center (HMC), a safety-net healthcare system in Seattle which is part of University of Washington (UW) Medicine—a large, integrated, academic-community practice. In 2017, through the UW Medicine Population Health Program, a quality improvement initiative to increase CRC screening through mailed FIT outreach was introduced. Between 2017 and 2019, ~11,000 UW Medicine patients received mailed FIT kits through the CRC screening initiative. Using a combination of approaches, including goal setting, leadership alignment, and mailed FIT outreach, CRC screening participation improved from 55 to 71% across UW Medicine. However, follow-up colonoscopy completion for patients with abnormal FIT results was suboptimal, especially within HMC where FIT uptake was high. Semi-structured interviews with HMC stakeholders revealed that lack of transportation is a significant patient barrier to follow-up colonoscopy completion (17). Transportation barriers were also confirmed by patients in a separate analysis (Issaka et al. unpublished).

For this study, we analyzed minutes from informal stakeholder engagement meetings that took place between August 2020 and August 2021 (*n* = 22 data collection points from *n* = 34 individuals). Informal stakeholder procedures are flexible and can include *ad-hoc* conversations to gather additional information as new topics emerge (21). This method was selected given the uncertainty of the COVID-19 pandemic at the time of study initiation. Meetings were scheduled with individuals or groups based on availability and COVID-19 safety precautions. First, the study senior author met with two health system medical directors to review existing policies on rideshare NEMT use. After confirming that existing policies could support rideshare NEMT for colonoscopy completion, we then engaged with other clinical and non-clinical stakeholders. Initial stakeholders were identified by the health system medical directors, gastroenterology leaders, and the research team. Interviewees were also invited to identify other relevant individuals and groups. The stakeholder meetings served as listening sessions to determine the barriers and facilitators to implementing a rideshare NEMT intervention for colonoscopy within our healthcare system.

Meetings were informal and did not use a pre-determined set of questions. Rather we used the information from our initial meeting and each stakeholder conversation to guide subsequent conversations. For example, we learned in our introductory meeting that a previous attempt to incorporate rideshare NEMT in the health system was unsuccessful due to a lack of agreement on contract terms between Social Work and the rideshare company. Thus, our conversations with Social Work focused on prior lessons learned that might inform our intervention. The listening sessions were not recorded, but we took thorough notes which were summarized and shared with stakeholders. The process recommendations for operationalizing the rideshare NEMT intervention were finalized using the nominal group technique, an evidence-based consensus methodology (22).

**Figure 1:**
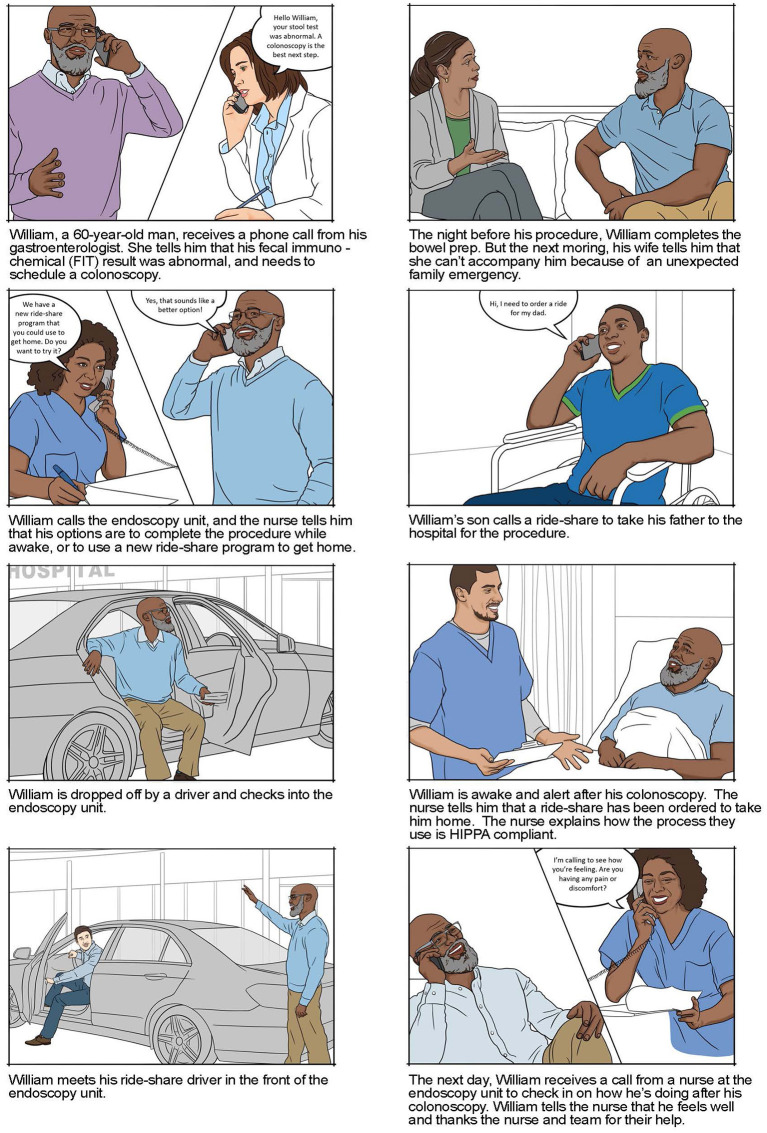
Sample storyboards used to guide stakeholder meeting.

Following the introductory meeting with health system medical directors, we convened a 2-h meeting with 10 stakeholders including members of the study team, health system medical directors, primary care and gastroenterology medical directors, nursing leaders, and rideshare industry representatives. We partnered with Lyft due to an existing relationship with a healthcare performance improvement company that contracts with Lyft and our health system. In this meeting, we used storyboards (Figure 1) to demonstrate real-world scenarios in which patients may benefit from a rideshare NEMT to complete a colonoscopy. Storyboards are a common technique for engaging users in the design process (21). We chose to use storyboards to ensure that all clinical and non-clinical stakeholders had a uniform understanding of the problem we hoped to address. The storyboards were developed by a medical illustrator based on real-world patient scenarios and determined to be appropriate by members of the study team, gastroenterology and primary care stakeholders. The meeting focused on determining the critical components needed to safely implement a rideshare NEMT program for colonoscopy completion, reviewed existing rideshare NEMT products, and confirmed policies that would prioritize patient safety. Topics that we would not have been able to discuss in depth if more time was needed to describe the clinical issue and how rideshare NEMT could serve as a viable solution.

After this meeting, the study team met separately with 24 additional health system stakeholders for 30–60-min video-conferences over a 6-week period. These stakeholders included representatives from Social Work, Risk Management and Compliance, Infection Prevention, the Endoscopy Suite, Contracting, and Anesthesia. Through our conversations with the health system stakeholders, we learned that the State Healthcare Authority (HCA) had a contract in place to provide NEMT for Medicaid patients and that any transportation provided to Medicaid patients would need to be conducted through the HCA. Thus, the study team also met with external partners from the State HCA and Hopelink (23) (a provider of NEMT for Medicaid patients in Washington State). Table 1 summarizes all the stakeholders who participated in this study.

**Table 1:** Key stakeholders and role in operationalizing a rideshare NEMT intervention.

**Title**	**Role in operationalizing rideshare NEMT**
Patient(s)	Identifies transportation as a barrier to colonoscopy and provides input on likelihood of using a rideshare NEMT
Primary care and ambulatory care medical director	Provides sign-off for any program being implemented in an ambulatory care setting
Gastroenterology medical director	Provides sign-off for program being implemented in gastroenterology
Chief of anesthesia clinical services	Helps determine which patient population is suitable for intervention based on procedural sedation
Chief of nursing	Ensures rideshare NEMT for patients receiving procedural sedation aligns with healthcare systems policies
Nurse manager of procedural unit	Provides input on the operationalization of a rideshare NEMT for patients receiving procedural sedation
Endoscopy business operations supervisor	Provides input on the operationalization of a rideshare NEMT for patients receiving procedural sedation
Patient care coordinators	Provides input on the operationalization of a rideshare NEMT for patients receiving procedural sedation
Endoscopy nurses	Provides input on the operationalization of a rideshare NEMT for patients receiving procedural sedation
Risk management and compliance	Provides sign-off stating that the risk of the program is acceptable for the healthcare system
Infection prevention	Due to COVID-19, any new program that puts a patient in contact with others needs to be approved by Infection Prevention
Social work	In our healthcare system, Social Work often coordinates transportation. They provided insight into existing transportation resources, past program negotiations, and how rideshare program may complement and fill gaps in existing resources
Health system contracting	Works with the rideshare organization to ensure the correct contracts are in place
Rideshare healthcare senior manager	Provides background and training on best platform for a rideshare program in a healthcare setting
Rideshare contracting	Works with healthcare organization to draft and execute a contract
State health care authority	May have existing contracts for NEMT programs for Medicaid patients
Mangers of healthcare system transportation vendors (if applicable)	Provides insight about the existing transportation options, volume of Gastroenterology rides provided, and opportunities for improvement

Immediately after each stakeholder meeting, two authors (ABB and RBI) met either in person or virtually to summarize the key take-aways and next steps. These summaries were updated based on feedback from participants, tracked in a shared document, and presented prior to subsequent stakeholder meetings. Our nominal group technique to reach consensus on how a rideshare NEMT could be operationalized within our healthcare system involved 5 participants (ABB, RBI, and three health system staff members). We initially all met in-person for 1 h, collectively drafted a workflow, and revised this iteratively in a “round robin” fashion. The final workflow was agreed upon by all 5 participants after several rounds of electronic and virtual communications and 2 in-person meetings with individuals who would execute the workflow (22). The study was approved by the Fred Hutchinson Cancer Research Center's Institutional Review Board.

## Results

### Barriers

#### Contractual Differences With Existing NEMT Services

NEMT is a benefit available for Medicaid beneficiaries to help overcome transportation barriers. In King County, Washington, where our healthcare system is located, the State Healthcare Authority (HCA) has an existing contract with a vendor to provide NEMT for Medicaid patients. Transportation companies that wish to provide NEMT for Medicaid patients must work with the HCA to have their vehicles dispatched through this vendor. However, such a partnership was not possible due to contractual differences between the HCA NEMT vendor and the rideshare NEMT vendor.

#### Lack of Door-to-Door Service

Within our healthcare system, the current Medicaid NEMT vendor provides “door-to-door” transportation service to patients who receive procedural sedation. In this setting, a driver picks up the patient from the procedural suite and brings them to the door of their destination. Current rideshare NEMT programs do not offer this service and instead offer a “curb-to-curb” service where patients are picked up at the hospital and dropped off in front of their home.

#### Inability to Modify Rideshare NEMT Programs

In our initial stakeholder meeting, we proposed two models for rideshare NEMT in settings where procedural sedation is administered. In one scenario, rideshare vendors would dispatch a select pool of drivers for curb-to-curb NEMT. In a second scenario, rideshare vendors would dispatch any available driver for door-to-door NEMT. However, we learned that existing rideshare NEMT programs could not be modified to meet institutional preferred practices such as door-to-door service or include only a select pool of drivers. This lack of flexibility may pose a significant barrier in some healthcare settings.

#### Lack of Chaperone

As previously described, rideshare NEMT only offers curb-to-curb NEMT, thus drivers are not required to go inside the healthcare facility to pick up patients. The largest rideshare companies, Lyft and Uber, both allow riders ~5 min to meet their driver in the designated pick-up location. In our health system, staff may accompany patients to their designated pick-up location, but this could potentially be an issue in health systems that don't allow staff to accompany patients.

### Facilitators

#### Cost

In a price comparison between Lyft and taxis offered through our healthcare system NEMT vendor, Lyft rides were on average 27% cheaper than taxis. As taxis are commonly booked for non-Medicaid patients unable to obtain rides following endoscopic procedures, and the costs billed to the healthcare organization, rideshare NEMT offers a potentially cost-saving solution in this population.

#### Shorter Wait Times

A persistent issue with the existing NEMT vendor is variable wait times for patient transportation. Pickup times are often unpredictable and if patients miss their window, it may take several hours to dispatch another vehicle. Using a rideshare NEMT program gives our healthcare system access to a larger fleet of vehicles, more readily available, within a shorter timeframe.

#### Existing Rideshare NEMT Platforms

The two major rideshare companies, Uber and Lyft, have existing NEMT platforms designed for patient transportation- Uber Health (24) and Lyft Concierge (25). Through these platforms, healthcare organizations can request rides for patients through a HIPAA compliant platform, bill the healthcare organization directly, and track patients to ensure that rides reach the specified destination. Both platforms can be operated by health system personnel and do not require patients own a smart phone for use.

### Process Recommendations for Operationalizing and Implementing a Rideshare NEMT Intervention

A contract between our healthcare system and Lyft Concierge^TM^ has been finalized and a pilot study to use rideshare NEMT for patients receiving colonoscopy is underway. The following process map may be useful to other healthcare systems that wish to implement a rideshare NEMT intervention in settings where procedural sedation is administered.

#### Identify Key Stakeholders

To explore the use of a rideshare NEMT program within our healthcare system, we first identified key stakeholders. These individuals/groups and their role in operationalizing a rideshare NEMT intervention are outlined in Table 1. While there may be differences between healthcare systems, ideally, these individuals/groups should have a firm grasp of institutional policies, past transportation programs or negotiations, and existing resources that may overlap with rideshare NEMT. In our study, we identified an initial group of stakeholders through the healthcare system leaders and discovered other important stakeholders through the listening sessions. Identifying these stakeholders early in the intervention implementation planning phase is a critical step (26).

#### Engage Each Stakeholder Group in Discussion to Develop the Rideshare Intervention Strategy

We then engaged representatives from each stakeholder group in discussions to operationalize a rideshare NEMT intervention in our healthcare system. These discussions included identifying barriers to rideshare NEMT for patients receiving procedural sedation and working with stakeholders to troubleshoot those barriers. Involving stakeholders in discussion around barriers and solutions allowed for a collaborative workflow development of the rideshare NEMT intervention and institutional buy-in.

#### Obtain Institutional Sign-Off

Identify individuals within the health system that will need to provide institutional approval to use rideshare NEMT for settings that administer procedural sedation. In our healthcare system, we required approval from primary care and gastroenterology medical directors, nurse managers, social work, risk management and compliance, and infection prevention prior to finalizing the partnership between the healthcare system and industry rideshare company.

#### Develop a Process for Reviewing and Selecting Available Rideshare NEMT Programs

Healthcare systems should consider any existing contracts or relationships with rideshare companies and if there is a rideshare NEMT product that could be immediately deployed for patient use. In our case, the connection between our healthcare system and Lyft through a third-party health performance improvement company, streamlined our process. We were able to modify an existing contract between our healthcare system and the healthcare performance improvement company to create a rideshare NEMT intervention for colonoscopy completion. Additional resources and time may be required if a partnership between a rideshare company and a healthcare system needs to be cultivated or if new rideshare NEMT products need to be created. Ideally, the rideshare NEMT program should allow rides to be booked by healthcare staff, comply with HIPAA regulations, and not require patients to own a smartphone as this may exacerbate disparities.

#### Execute Contracts

A final contract should be executed between the rideshare company and the partnering healthcare system. We executed a final contract between the healthcare system and the rideshare company which outlines the rideshare NEMT services offered, agreement term limits, patient safety policies (which should include minimizing the spread of COVID-19 during the pandemic), insurance coverage and liability. If Medicaid patients will be included in the rideshare NEMT program, a separate contract may be required by the state's healthcare authority.

#### Develop a Standard Operating Procedure for Executing the Rideshare Intervention Strategy

Working collaboratively with endoscopy personnel, we developed a standard operating procedure for staff use. This workflow details, step-by-step, how eligible patients will be identified and notified of the rideshare NEMT program, how and where to document when patients use the rideshare program, and when staff escorts would be used. In our system, patients will need to provide informed consent and agree to participate in semi-structured interviews in the days following their procedure to assess the safety, feasibility, acceptability, and appropriateness of the rideshare intervention strategy (27). Endoscopy staff will also be interviewed to determine similar outcomes.

#### Train Clinic Staff on Use of Rideshare Platform

Endoscopy personnel should receive training on using the rideshare platform including how to reserve rides, how to track rides to ensure patients are dropped off at the correct location, and how to pull facility reports for hospital administration quality control.

## Discussion

In this study, we identified barriers, facilitators, and process to implementing a rideshare NEMT intervention for colonoscopy completion in a safety-net healthcare setting. Our findings informed a cross-sector partnership between a healthcare system and industry to pilot an intervention to improve access to transportation after procedural sedation, a common barrier to CRC screening, especially for racial/ethnic minorities and low-income populations. This partnership directly aligns with Public Health 3.0's recommendation to address social determinants of health (SDOH) (28).

Transportation is one of many SDOH that contributes to inadequate preventive care including CRC screening. SDOH are the conditions in the environments where people are born, live, learn, work, worship and age that affect a wide range of health, functioning, and quality of life (29). In this manuscript, we detail the process of operationalizing a rideshare NEMT for colonoscopy completion which to date has included 22 data collection points from 34 stakeholders and spanned over a year. Indeed, opportunities to address transportation and other SDOH through cross-sector partnerships should be pursued whenever possible, but the importance of government directed health policy that addresses SDOH cannot be overstated. For example, universal health insurance that includes transportation benefits could substantially reduce transportation barriers in CRC screening and other preventive diseases, especially for lower-income, rural, and other under-resourced populations.

NEMT is a mandatory benefit for Medicaid and some Medicare Advantage enrollees, but transportation remains a persistent barrier for patients in safety-net health systems and FQHCs due to lower incomes, underinsurance, and lack of insurance (30). Transportation requirements for colonoscopy completion may be especially challenging for safety-net patients who experience fragmented care due to complex medical conditions and social circumstances (12, 17). Indeed, there is a need for NEMT options for patients whose health plans lack a mandatory benefit. In prior research, non-rideshare NEMT as part of patient navigation programs, increased colonoscopy completion compared to controls (31), was cost-effective (32), and improved patient satisfaction (16). To our knowledge, ours is the first study to describe the barriers, facilitators, and process of operationalizing a rideshare NEMT intervention in a safety-net population after procedural sedation for colonoscopy completion.

Rideshare NEMT is a potentially scalable and cost-effective intervention but has not been optimized for settings that administer procedural sedation. In this study, we define the process steps for operationalizing a rideshare NEMT intervention for colonoscopy completion in a safety-net healthcare system. By doing so, we add contextual data that is frequently missing from intervention implementation but is critical for translating research findings to real-world improvements in healthcare outcomes (33). Thus, our findings could lead to increased colonoscopy completion in safety-net health settings and other settings that provide care to medically underserved populations and have broad implications for other locations where procedural sedation is administered (e.g., bronchoscopy suites).

Our study has limitations. First, we interviewed key stakeholders knowledgeable about system-level policies and the complexity of colonoscopy completion. It is possible that the barriers, facilitators, and outlined process may have differed in a different group of stakeholders. Second, due to the climate in which our study began, our interviews were not recorded or transcribed verbatim and this could have biased our conclusions. Third, our study was conducted in an urban safety-net system and these findings may not be generalizable to different settings. We believe these limitations are offset by the rigor of our scientific approach which included an iterative process of stakeholder input and the nominal group technique, the novelty of the resultant cross-sector partnership, and the unique opportunity to address a significant SDOH.

To improve CRC screening through initial and follow-up colonoscopy completion, multi-component interventions are needed (34). Our prior work and other published research have identified multiple barriers and facilitators to colonoscopy completion at the patient, provider, and health-system level for which there are practical solutions (17, 35, 36). In this paper, we outline the process of operationalizing a rideshare intervention for colonoscopy completion in a safety-net population. In addition to addressing transportation barriers, determining the combination of interventions that will most effectively improve colonoscopy completion in CRC screening is an important next step.

## Data Availability Statement

The datasets presented in this article are not readily available because participants did not consent to publicly sharing generated data. Requests to access the datasets should be directed to Rachel B. Issaka, rissaka@fredhutch.org.

## Ethics Statement

The studies involving human participants were reviewed and approved by the Fred Hutchinson Cancer Research Center/University of Washington Cancer Consortium's Institutional Review Board. Written informed consent for participation was not required for this study in accordance with the national legislation and the institutional requirements.

## Author Contributions

RI had full access to all the data in the study and takes responsibility for the integrity of the data and the accuracy of the data analysis, obtained funding, conceptualized, designed, acquired, analyzed, and interpreted the data from stakeholders and was a major contributor in writing the final manuscript. AB-B acquired, analyzed, and interpreted the data from stakeholders and was a major contributor in writing the final manuscript. LC, LS, and BB were stakeholders who informed the design of the study. BW, PH, and CL obtained funding for this study and provided critical revision of the manuscript for important intellectual content. JI and SR provided critical revision of the manuscript for important intellectual content. All authors read and approved the final manuscript.

## Funding

Research reported in this publication was supported by the National Cancer Institute of the National Institutes of Health under Award Number K08CA241296 (RI) and Award Number P50CA244432 (BW, CL, PH, and RI).

## Conflict of Interest

RI reported receiving grants from National Institutes of Health/National Cancer Institute award number K08 CA241296 and P50CA244432 for the conduct of this study. BW, CL, and PH reported receiving grants from National Institutes of Health/National Cancer Institute award number P50CA244432 for the conduct of this study. The remaining authors declare that the research was conducted in the absence of any commercial or financial relationships that could be construed as a potential conflict of interest.

## Publisher's Note

All claims expressed in this article are solely those of the authors and do not necessarily represent those of their affiliated organizations, or those of the publisher, the editors and the reviewers. Any product that may be evaluated in this article, or claim that may be made by its manufacturer, is not guaranteed or endorsed by the publisher.
